# GenAI-Supported Virtual Patients in Health Care Education: Systematic Review

**DOI:** 10.2196/82756

**Published:** 2026-05-07

**Authors:** Juming Jiang, Megan Zichen Ye, Tyrone Tai-On Kwok, Janet Yuen Ha Wong

**Affiliations:** 1 School of Nursing and Health Sciences, Jockey Club Institute of Healthcare, Hong Kong Metropolitan University, 11th Floor, 1 Sheung Shing Street, Homantin, Kowloon, Hong Kong, China (Hong Kong), 852 39702988

**Keywords:** systematic review, generative AI, virtual patient, health care education, PRISMA, generative artificial intelligence, Preferred Reporting Items for Systematic Reviews and Meta-Analyses

## Abstract

**Background:**

Generative artificial intelligence (GenAI) is enhancing virtual patient simulations in health care education by enabling dynamic, adaptive interactions, reshaping how clinical skills are taught. A synthesis of the current evidence is needed to guide implementation and future research, given the pace of technological advancement.

**Objective:**

This systematic review aims to synthesize empirical research on the design, implementation, and educational impact of GenAI-supported virtual patients in health care education.

**Methods:**

A systematic search was conducted across 5 databases (CINAHL, Medline, Embase, Scopus, and Web of Science) from their inception to March 19, 2026. Reference lists of included studies and relevant systematic reviews were also screened. Peer-reviewed studies in English that evaluated GenAI-supported virtual patients using quantitative or mixed methods were included. Two reviewers independently screened studies and extracted data. Study quality and risk of bias were assessed critically using JBI (Joanna Briggs Institute) checklists, with disagreements resolved by consensus.

**Results:**

A total of 15 studies met the inclusion criteria (total participants N=645), spanning health care disciplines, including nursing, medicine, pharmacy, radiography, and medical first-responder training. The virtual patients varied in design; input modalities included text (9 studies), voice (5 studies), or hybrid (1 study); output was text (9 studies), speech (5 studies), or both (1 study); 6 studies used 3D-embodied avatars, while 9 used nonembodied interfaces. A total of 13 studies used OpenAI GPT models (eg, ChatGPT), 1 used a fine-tuned model from a different provider, and 1 evaluated multiple model families (Claude, GPT, and open-source). Further, 6 studies used controlled experimental designs, including 3 randomized controlled trials (RCTs); the remainder were cross-sectional or prepost evaluations. Primary outcomes included user perceptions (14 studies), communication skills (4 studies), clinical reasoning (3 studies), and performance (7 studies). In controlled comparisons, GenAI-supported virtual patients consistently improved outcomes relative to control conditions: for example, enhanced clinical decision-making (RCT, n=21), ophthalmology history-taking skills (RCT, n=26), and medical history-taking performance (crossover RCT, n=20). The evidence base is characterized by brief intervention durations, a predominant reliance on single-session interactions, and a general lack of underpinning educational theory. No meta-analysis was performed due to the limited number of studies and significant heterogeneity in designs, interventions, and outcome measures.

**Conclusions:**

The evidence supports the feasibility and acceptability of GenAI-supported virtual patients, with positive learner perceptions and promising outcomes for skills development. However, critical limitations remain in emotional-behavioral complexity, simulation adaptability, and research design rigor (eg, limited use of control groups and validated instruments). The review offers educators, instructional designers, and policymakers actionable insights for integrating dynamic, artificial intelligence–driven simulations while identifying crucial gaps—such as the need for theoretical grounding, longitudinal studies, and standardized design protocols—that must be addressed for safe and effective implementation.

## Introduction

### Background

Virtual patients are sophisticated educational simulations designed to replicate authentic clinical scenarios, enabling health care trainees to practice skills in a safe, controlled environment without risk to real patients [1,2]. They are defined as “a representation of an actual patient,” which can include various forms such as software-based simulators or manikins, and specifically as “a computer program that simulates real-life clinical scenarios in which the learner acts as a health care provider,” making clinical decisions [3]. They serve as a cornerstone of modern clinical education, aiming to enhance clinical reasoning, communication proficiency, and decision-making abilities [4]. The recent and rapid integration of generative artificial intelligence (GenAI)—a subset of artificial intelligence (AI) powered by large language models (LLMs) and natural language processing—is fundamentally transforming this educational tool [5,6]. Unlike traditional scripted simulations, GenAI-supported virtual patients can generate dynamic, adaptive, and contextually relevant responses in real-time, allowing for more realistic conversations, emotional responsiveness, and personalized learning experiences [7,8]. This shift marks a significant evolution in simulation-based learning, moving from static, preprogrammed cases toward interactive, intelligent patient encounters.

### Rationale

The evolution of virtual patients demonstrates a clear trajectory toward greater interactivity and realism. From early, static computer-based cases on systems such as PLATO [9], modern iterations now incorporate multimedia, branching narratives, and data-driven models to create more engaging and realistic clinical encounters [10,11]. This evolution has yielded significant educational benefits. Empirical studies show that virtual patients can improve clinical decision-making, diagnostic accuracy, and foundational skills such as screening and referral across various health disciplines [11,12]. For instance, they have been successfully implemented as standardized, unfolding simulations to replace scarce pediatric clinical hours while maintaining clinical competency in nursing education [13]. Furthermore, a pilot study grounded in Experiential Learning Theory demonstrated that virtual patient simulation led to statistically significant improvements in clinical reasoning and communication skills among prelicensure nursing students [14]. They provide a scalable, consistent training environment that addresses limitations inherent to human standardized patients, such as fatigue and variability [7,15].

Despite these advances, a critical constraint remains: the predominant reliance on prescripted, linear scenarios. This often results in predictable interactions that fail to fully replicate the dynamic, adaptive, and complex nature of real patient encounters, potentially limiting learner engagement and the ability to respond to individualized student input [16,17]. Consequently, there is a recognized need for more dynamic and adaptable virtual patient models to better prepare learners for clinical practice [18].

GenAI presents a transformative solution to this limitation. A subset of AI powered by LLMs, GenAI can generate context-aware, realistic text, dialog, and even simulate human behaviors in real-time [19,20]. In health care education, integrating GenAI allows virtual patients to move beyond scripts, generating unique, adaptive responses based on learner inquiries and simulating a wider range of patient behaviors and emotional states [7,8]. This capability promises to significantly enhance the authenticity, personalization, and educational value of simulation-based training.

However, the implementation of this novel technology introduces new complexities regarding its design, pedagogical integration, and evaluation. While systematic reviews have established the effectiveness of traditional, scripted virtual patients [21,22] and scoped the broad potential of GenAI in education [5], a critical gap remains. Existing syntheses are unequipped to address the unique research questions (RQs) generated by their convergence. Specifically, the literature lacks evidence-based guidance on how the adaptive, nondeterministic nature of GenAI alters optimal instructional design, what novel evaluation frameworks are required to measure its impact on dynamic clinical reasoning, and how the practical challenges of implementation differ from those of static simulations. This dedicated synthesis is absent. Consequently, without a focused systematic review, evidence regarding the optimal design features, verifiable educational impact, and practical challenges of these advanced tools remains fragmented, hindering evidence-based adoption and focused research development [23,24].

Given these factors, it is essential to conduct a systematic review that not only summarizes the characteristics of GenAI-supported virtual patients but also provides comprehensive guidance for future research in this emerging field. Such a review will help educators, researchers, and policymakers understand the potential benefits and limitations of GenAI-supported virtual patients, thereby facilitating their integration into health care education curricula.

### Objectives

The primary objective of this systematic review is to synthesize the current empirical evidence on the design, implementation, and educational impact of GenAI-supported virtual patients in health care education. Specifically, the review addresses the following RQs:

RQ1. What are the design choices, technological architecture, and educational strategies embedded within GenAI-supported virtual patients?

RQ2. What are the evaluation and educational impact, including benefits, outcomes, and related limitations of GenAI-supported virtual patients?

## Methods

### Study Design

This review was conducted and reported in accordance with the PRISMA (Preferred Reporting Items for Systematic Reviews and Meta-Analyses) 2020 statement and its expanded checklist (Checklist 1) [25], as well as synthesis without meta-analysis (Multimedia Appendix 1) [26]. The protocol was registered and published in the Open Science Framework repository [27].

### Eligibility Criteria

The following inclusion criteria were used: (1) original studies published in peer-reviewed journals, (2) focused on evaluating how GenAI-supported virtual patients affect education and training in health care–related disciplines, (3) included measurements of user experience or user outcomes, (4) conducted using quantitative or mixed methods research design, and (5) written in English. The exclusion criteria were the following: (1) book chapters, editorials, short communications, letters, and review literature; and (2) studies focused solely on the technical development of virtual patients without educational evaluation.

### Information Sources

A systematic search was performed across 5 databases from their inception to March 19, 2026: CINAHL (via EBSCOhost), MEDLINE (via EBSCOhost), Embase (via Elsevier), Scopus (via Elsevier), and Web of Science Core Collection (via Clarivate). These databases were chosen for their comprehensive coverage of biomedical, nursing, allied health, and interdisciplinary literature. Furthermore, the reference lists of all included full-text papers were manually reviewed to identify additional relevant studies.

### Search Strategy

The search strategy was developed iteratively for each database to ensure comprehensive coverage of the core concepts of “virtual patient” and “generative artificial intelligence.” For each database, the strategy combined controlled vocabulary (where available, eg, MeSH [Medical Subject Headings] in MEDLINE and CINAHL Subject Headings) with extensive free-text terms, using appropriate field-specific syntax (eg, MH and XB in CINAHL and MEDLINE, :ti,ab in Embase, TITLE-ABS-KEY in Scopus, and TS= in Web of Science). Synonyms for virtual patients and GenAI models were grouped logically, and all free-text terms were searched in the title, abstract, and keyword fields. Searches were executed using the advanced search interface of each platform. The search was limited to records published in English and, where available, to peer-reviewed papers. The search was updated and rerun on March 19, 2026, after incorporating expanded terms and controlled vocabulary as suggested during peer review. The full search strategy is reported in Multimedia Appendix 2 and in accordance with the PRISMA-S (Preferred Reporting Items for Systematic Reviews and Meta-Analyses Literature Search Extension) checklist [28].

Several elements of the PRISMA-S checklist [28] did not apply to our methodology: we did not search databases simultaneously on a single platform (item 2); we did not search study registries (item 3); we did not browse online or print sources (item 4); we did not perform citation searching beyond checking reference lists of included studies (item 5); we did not contact authors, experts, or manufacturers to identify additional studies (item 6); we did not use any other information sources or methods beyond those described (item 7); we did not use published search filters (item 10); we did not adapt search strategies from prior reviews (item 11); we did not set up email alerts or automated updates, but we did manually rerun the search after refining the strategy (item 12); and the search was not formally peer reviewed (item 14). These items are explicitly noted as not applicable in this paper.

### Selection Process

All records identified from the database searches were imported into Zotero reference management software for deduplication. The selection process was conducted in 2 phases. First, 2 reviewers (JJ and MZY) independently screened the titles and abstracts of all records against the eligibility criteria. Second, the full texts of potentially eligible studies were retrieved and independently assessed for inclusion by the same 2 reviewers. Any disagreements at either stage were resolved through discussion between the reviewers until consensus was reached. The interrater reliability was calculated using Cohen κ, resulting in κ=0.7, which indicates substantial agreement [29].

### Data Collection Process

A standardized data extraction form was developed in Google Sheets (Google LLC). Further, 2 reviewers (JJ and MZY) independently extracted data from each included study. The extracted data were then cross-checked, and any discrepancies were resolved through discussion. The interrater reliability was κ=0.6, which indicates moderate agreement [29].

### Data Items

Google Sheets (Google LLC) were used for data extraction. The following information was extracted from each of the papers that met the inclusion criteria: (1) publication characteristics: authors, publication year, and source; (2) study characteristics: study design and sample size; (3) intervention characteristics: description of the GenAI-supported virtual patient, including input or output modalities, use of an avatar, duration of interaction, technological details (eg, GenAI model and prompt engineering), and integration with educational strategies or theories; (4) outcomes: all reported outcome measures, including primary and secondary outcomes related to user perceptions (eg, usability, satisfaction, and perceived learning), skills (eg, communication and clinical reasoning), and performance; and (5) key results: main quantitative and qualitative findings as reported by this study’s authors.

### Study Risk of Bias Assessment

To critically appraise the methodological quality and risk of bias of the included studies, a formal assessment was conducted in accordance with PRISMA guidelines (item 11). The JBI (Joanna Briggs Institute) critical appraisal checklists, appropriate to each study design, were used as standardized tools [30]. Specifically, the JBI Checklist for Randomized Controlled Trials was used for randomized controlled trials (RCTs) [31], the JBI Checklist for Quasi-Experimental Studies was used for nonrandomized comparative studies [32], and the JBI Checklist for Analytical Cross-Sectional Studies was used for cross-sectional evaluations [32]. Further, 2 reviewers independently assessed each study. Any discrepancies in appraisal judgments were resolved through discussion to reach consensus. The interrater reliability was κ=0.8, which indicates substantial agreement [29]. The results of this assessment are synthesized narratively and were considered when interpreting the overall strength and validity of the evidence presented in this review.

### Effect Measures

Current review synthesized findings narratively, thus no common effect measures were pooled across studies due to significant heterogeneity in study designs, interventions, and outcome measures.

### Synthesis Methods

A meta-analysis was not feasible due to the limited number of studies and substantial heterogeneity in interventions, populations, and outcome measures. Therefore, a narrative synthesis was conducted. The extracted data were summarized and organized to address the review’s RQs. Findings were structured thematically to describe: (1) the implementation characteristics (design, technology, and educational strategies) of GenAI-supported virtual patients, and (2) their evaluation and educational impact (benefits, outcomes, methodological approaches, and limitations). The synthesis also integrates a discussion of the methodological quality and risk of bias of the included studies.

### Reporting Bias Assessment

No formal statistical assessment of publication bias (eg, funnel plot) was performed due to the narrative synthesis approach and the small number of included studies.

### Certainty Assessment

A formal assessment of the certainty of the body of evidence (eg, GRADE [Grading of Recommendations, Assessment, Development, and Evaluation]) was not conducted for this narrative review, as its primary aim was to map and characterize an emerging field rather than to estimate a pooled treatment effect.

## Results

### Study Selection

**Figure 1.**
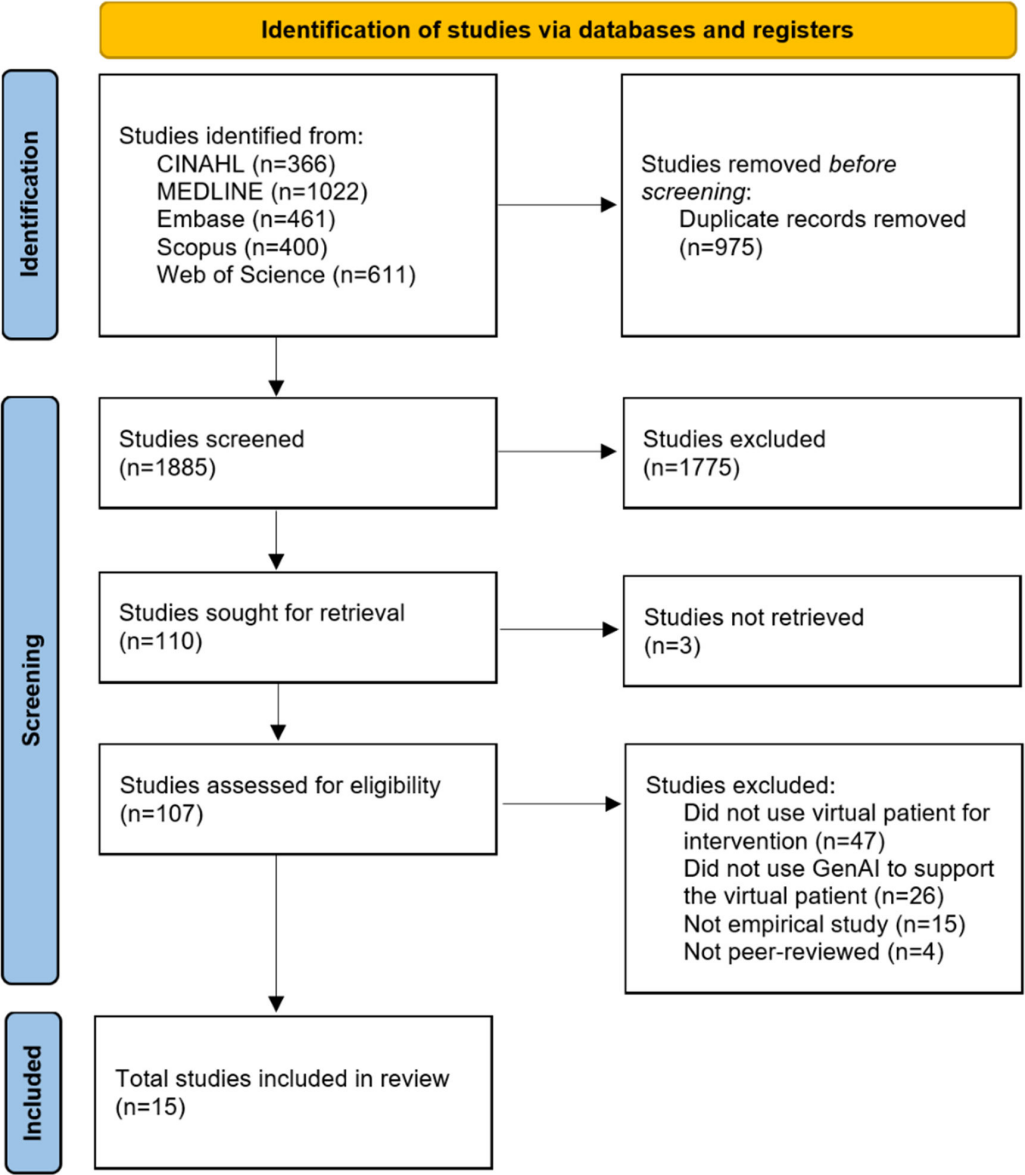
Summary of the selection of publications. GenAI: generative artificial intelligence.

The initial literature search yielded 2860 studies. After screening the abstracts, 107 papers were selected for further evaluation. Further, 2 authors screened full texts independently, and the screening was then discussed as a group to ensure consensus and make the final selection decision. Ultimately, 15 papers were included in the final analysis after reading the full-text. Figure 1 provides a detailed overview of the systematic search procedure.

### Study Characteristics

All 15 studies included in this review were published in peer-reviewed journals. Research in this area was prominently featured in the JMIR portfolio, which published 6 of the included papers: 3 papers in *JMIR Medical Education* [33-35], 2 papers in the *Journal of Medical Internet Research* [36,37], and 1 paper in *JMIR Formative Research* [38]. Temporally, 8 studies were published in 2024 [33-35,38-42], marking a peak of initial investigative activity, followed by 7 more studies in 2025 and 2026 [36,37,43-47], indicating sustained scholarly interest. The included studies used a diverse range of methodologies. The 2024 cohort predominantly featured cross-sectional (n=5) [33,34,38,39,42] and quasi-experimental (n=2) [35,40] designs, alongside 1 RCT [41]. The 2025‐2026 publications demonstrated a continued diversification of methods, including 2 additional RCTs [45,47], 4 quasi-experimental studies [36,43,44,46], and 1 proof-of-concept observational study [37]. Participant sample sizes varied widely, from smaller feasibility studies (eg, n=6 [40]) to larger-scale evaluations (eg, n=145 [35,43]), and a paired crossover study with 20 medical students [47]. The duration and frequency of interventions with the GenAI-powered virtual patients also differed, ranging from brief, single interactions of approximately 6‐10 minutes [38] to more extended or repeated practice sessions over several weeks [43]. This heterogeneity in publication venues, design, scale, and intervention format reflects the exploratory and rapidly evolving nature of research in this domain.

Moreover, among the 15 included studies, 13 of them explicitly stated obtaining ethics approval from an institutional review board and described informed consent procedures [33-39,41,43-45]. Specific data privacy or security measures were reported less consistently, detailed in only 8 (54%) studies [33-35,37-39,43,46]. Notably, 2 studies [40,42] documented that formal ethics approval was not required for their projects, citing their design as a quality assurance activity and a survey of professionals, respectively.

### Risk of Bias in Included Studies

The methodological quality of the 15 included studies, as assessed by the JBI critical appraisal checklists, demonstrated a spectrum of risk of bias (Table 1). Further, 3 RCTs were judged to have a low to low-moderate risk of bias [41,45,47], benefiting from strong methodologies including randomization, allocation concealment, and blinded outcome assessment. However, the nature of the interventions precluded participant blinding, a common limitation in educational technology studies. In contrast, the body of quasi-experimental [35,36,40,43,44,46] and cross-sectional studies [33,34,37-39,42] presented a higher risk of bias. Common methodological weaknesses across these designs included the use of small, nonrepresentative samples, a lack of control groups, the absence of preintervention or longitudinal outcome measurements, and frequent reliance on unvalidated or self-reported outcome measures. Consequently, while the evidence from RCTs provides a stronger foundation for causal inference regarding the impact of GenAI-supported virtual patients, the overall findings of this review—particularly those related to user perceptions and feasibility—are drawn from a predominantly moderate-risk evidence base. This necessitates a cautious interpretation of the results, as positive outcomes may be influenced by study design limitations and enthusiasm for a novel technology.

**Table 1.** Risk of bias assessment of included studies.

Study type and reference citation	Overall risk of bias or appraisal judgment	Key concerns or limitations
RCTs^a^		
[41]	Low to moderate risk	Unclear allocation concealment; potential lack of blinding for treatment deliverers.
[45]	Low risk	Lack of participant blinding (acknowledged as an expected limitation for the intervention type).
[47]	Low risk	Well-designed crossover RCT; lack of participant blinding is an inherent limitation.
Quasi-experimental studies		
[40]	Moderate to high risk	Intentional selection of dissimilar participants; no control group; no preintervention measurement; unclear reliability of qualitative coding.
[35]	Moderate risk	Nonrandomized historical control group; no pretest for primary outcome; intervention was supplementary to the standard curriculum.
[36]	Moderate risk	Lack of a preintervention baseline measure for the primary outcome.
[43]	Moderate risk	No control group; no pretest measurement; reliance on self-reported data; unclear reliability of survey instrument.
[44]	Moderate risk	No control group; only partial prepost measurement for some outcomes; small convenience sample.
[46]	Moderate risk	Between-groups design with nonrandomized assignment; small sample size; single-site study.
Analytical cross-sectional studies		
[39]	High risk	Very small sample; lack of validated measures; no control for confounders; limited generalizability.
[33]	Moderate risk	Lack of confounding factor consideration; use of a single case scenario.
[34]	Moderate risk	Lack of consideration for confounding factors; single case or model; potential selection bias.
[42]	Moderate risk	Small sample; lack of objective outcome measures; no control group; potential for selection or response bias.
[38]	Moderate risk	Subjective outcome measures; no objective performance assessment; no control group; small sample.
[37]	Moderate risk	No control group; small single-site sample; reliance on self-reported benefits; no long-term skill retention measures.

a RCT: randomized controlled trial.

### Results of Syntheses

To address the RQ1 concerning the design and implementation characteristics of GenAI-supported virtual patients, the following sections analyze the extracted data, which are comprehensively tabulated in Table 2.

**Table 2.** Characteristics of the virtual patients and the GenAI^a^ models used in the 15 included studies. Detailing input or output modalities, avatar design, the AI’s^b^ tasked role, and using educational frameworks.

Reference citation	Input (eg, text and voice)	Output (eg, text and voice)	Avatar (2D/3D)	Movement	Emotion expression	Type or name of AI	Overview of the task for GenAI based on the designated prompt	Educational theories or models
[39]	Text	Text	N/A^c^	N/A	N/A	ChatGPT (version not specified)	Simulate a realistic clinical interaction focusing on assessment, communication, and nursing care for respiratory distress	N/A
[40]	Text	Text	N/A	N/A	Using case scenarios such as patients with claustrophobia undergoing an MRI^d^ scan	OpenAI ChatGPT 3.5 and ChatGPT 4	A roleplay designed to simulate a conversation between a radiology technician and a patient with claustrophobia during an MRI examination. The AI takes the role of the patient, while the user plays the technician.	N/A
[33]	Text	Text	N/A	N/A	N/A	OpenAI GPT-4	Two prompts were developed: one for providing the interactive history-taking dialog, and the other for giving feedback	N/A
[34]	Text	Text	N/A	N/A	N/A	OpenAI GPT-3.5	GPT acts as a simulated patient. The prompts were designed to guide GPT’s behavior and ensure it provided medically accurate and relevant responses.	N/A
[35]	Text	Text	N/A	N/A	The emotional parameters were set from 1 to 10 for 8 emotions: joy, sadness, anticipation, surprise, fear, disgust, trust, and anger	GPT-4 Turbo	The prompt is designed to simulate a chatbot role-playing as a medical patient with dynamic emotional behavior. It consists of two major phases: (1) roleplay phase (simulated patient behavior): governs how the chatbot behaves during the medical consultation, and (2) feedback phase (interaction evaluation): after the roleplay ends, the chatbot switches to feedback mode and evaluates the user’s performance.	N/A
[41]	Text	Text	N/A	N/A	N/A	OpenAI GPT-3.5	Control group (AI simulation only): a virtual patient scenario crafted for emergency and neurological assessment training. The AI simulates a patient experiencing a traumatic brain injury. Feedback group (AI simulation+ AI feedback): the AI first simulates the patient with the same setting in the control group and then provides diagnostic feedback assessment for participants who play as the doctor after their interaction.	N/A
[42]	Text, voice	Text, voice	3D	N/A	N/A	Generative conversational AI, specifically using the platform Convai (Convai Technologies Inc) and incorporating ChatGPT (version was not mentioned) for text generation	Provide comprehensive content on ORIF^e^ surgery suitable for training a large language model, which is then subsequently further expanded.	N/A
[38]	Voice	Voice	3D	Vive Trackers (version 3.0) were placed on the head, hands, feet, and groin of the manikin and mapped to the corresponding parts of the VP’s^f^ avatar. This allowed the MFRs^g^ to freely move the manikin and thus the VP.	Emotion is expressed through the voice, including stuttering, groans, and cries, as well as statements reflecting fear and pain	OpenAI GPT-3.5-Turbo	This simulation helps practice trauma-informed care, nonverbal cues, and managing patients in acute distress. All responses are limited to a maximum of 8 stuttered words.	N/A
[36]	Voice (student’s spoken questions, converted to text via speech-to-text)	Voice (synthesized speech via text-to-speech) Visual (facial expressions projected onto the robot)	3D (Furhat social robot with animated face back-projected onto a translucent mask)	Natural head movements (neck with 3 degrees of freedom)	Facial expressions (eg, sad, happy, or surprised) were generated and synchronized with speech	OpenAI GPT-3.5-turbo	Dialog generation: to generate the next patient dialog line. The prompt includes the patient case description, the last 10 dialog turns, and instructions to respond as the patient. Expression generation: to select appropriate facial expressions (from a predefined set) at anchor points within the generated dialog text to reflect the patient’s emotional state.	N/A
[43]	Text	Text	N/A	N/A	Textual descriptions of emotional state within dialog (eg, expressing stress or motivation). No visual or auditory emotion simulation	OpenAI ChatGPT 3.5	Patient simulation: to act as a smoker seeking to quit, responding in character to student-led counseling based on a predefined case scenario that includes demographics, smoking habits, and motivations. Performance feedback: after the counseling session, to evaluate the student’s performance based on a structured rubric (the 5As^h^ framework, empathy, communication skills, etc) and provide detailed textual feedback on strengths and areas for improvement.	The 5As framework for smoking cessation counseling
[37]	Text	Text	N/A	N/A	Textual descriptions of the patient’s emotional state within dialog (eg, expressing anxiety or distress). No visual or auditory emotion simulation	OpenAI GPT-4	Patient simulation: to act as a simulated patient (eg, a man aged 28 years with depression or a woman aged 46 years with agoraphobia) and respond in character to physician-led text-based dialog. Real-time feedback generation: to analyze the physician’s incoming text messages in real-time, identify the use of specific communication techniques (eg, open questions, reflections, empathy, or validation), and provide immediate formative textual feedback within the chat to confirm and encourage technique use. Summative feedback generation: after the chat, to provide summarized feedback on the frequency of technique use, highlight underused techniques, and give examples for future application.	N/A
[44]	Voice (learner’s spoken questions to the virtual patient via HMD^i^, processed by a speech-to-text model)	Voice (virtual patient’s spoken responses generated by the AI, delivered via HMD with an AI-generated voice)	3D	Limited (the virtual human avatar is present but has limited physical interaction; cannot perform actions such as raising clothes or turning over as requested)	Limited (primarily text-based emotional cues within dialog. This study notes limitations such as unnatural voice expressiveness and an absence of emotional sentiment	OpenAI GPT-4o	Patient simulation: to act as a virtual patient (a male aged 28 years with acute appendicitis) and engage in real-time, natural language dialog with nursing students for health assessment and communication training. The AI is provided with a detailed patient script and vital sign data to generate contextually relevant and medically accurate responses to the learner’s verbal questions.	Instructional design model for AI education Technology acceptance model (for evaluation)
[45]	Voice	Voice	Visual avatar (image of patient’s eye; 2D/3D not specified)	N/A	Expressed through voice (eg, anxiety or emotional responses)	Fine-tuned Baichuan-13B-Chat (a large language model)	To simulate a digital ophthalmology patient for medical history-taking practice. The AI acts as the patient, responding in character to students’ verbal inquiries based on a detailed knowledge base derived from electronic health records. The system provides real-time interaction and, after the session, generates automated feedback and scores based on the comprehensiveness of the history taken.	Kolb’s experiential learning cycle Calgary-Cambridge communication framework
[46]	Voice	Voice	3D	Not specified (avatar is static in the examination room; no description of physical movement)	Facial expressions rendered in real-time by the D-ID^j^ platform to create a full audiovisual experience; tone of voice (eg, irritable or rapport-building) also conveys emotion	OpenAI GPT-4o (via API^k^ calls)	Patient simulation: to act as a virtual patient (“Randy Rhodes,” a man aged 54 years with type 2 diabetes) for medical students to interview via voice-to-voice interaction. Custom agent instructions are informed by faculty-generated case materials. “Guardrails” are placed to optimize educational value (eg, preventing the AI from revealing the diagnosis directly or ensuring accurate presentation of pertinent positive findings). Within these limits, the AI is allowed to respond adaptively to students’ questions to maintain realism.	N/A
[47]	Text	Text	N/A	N/A	Textual descriptions of patient emotional state are generated based on integrated personality profiles (eg, the Big Five framework) to simulate emotional realism (eg, pain and anxiety). No visual or auditory emotion simulation	Claude models (5): Claude3 Haiku Claude-3-Sonnet Claude-3‐5 Sonnet Claude-4-Sonnet Claude-4-Opus GPT-family models (3): GPT-4 Turbo GPT-4o GPT-3.5 Turbo Open-source models (3): DeepSeekV3 671B Qwen3-32B LLaMa-3 70B	To act as a simulated patient (“AIPatient”) based on real EHR^l^ data from the MIMIC-III^m^ database. The AI engages in text-based dialog with medical students for history-taking practice. Its task is to provide accurate, readable, and consistent responses to clinical questions while incorporating diverse personality traits to simulate realistic patient behavior, including emotional expressions.	N/A

a GenAI: generative artificial intelligence.

b AI: artificial intelligence.

c N/A: not applicable.

d MRI: magnetic resonance imaging.

e ORIF: open reduction and internal fixation.

f VP: virtual patient.

g MFR: medical first responder.

h 5A: Ask, Advise, Assess, Assist, Arrange.

i HMD: head-mounted display.

j D-ID: deidentification.

k API: application programming interface.

l EHR: electronic health record.

m MIMIC-III: Medical Information Mart for Intensive Care III.

### Design Choices

The designs of GenAI-supported virtual patients can be classified into three distinct categories: (1) input, (2) output, and (3) avatar. First, in terms of input methods, 11 of 15 studies reported that participants interacted with the virtual patient by entering text [33-35,37,39-41,43,47]. Meanwhile, 5 studies allowed participants to communicate verbally using voice input [36,38,44-46]. Additionally, 1 study incorporated a hybrid approach, enabling participants to use either speech-to-text functionality via a microphone or direct text input through a designated field [42]. Next, the output modalities of the virtual patient corresponded closely to the input mechanisms. In 9 studies, the virtual patient responded to participants via text-based communication [33-35,37,39-41,43,47]. In contrast, 5 studies featured virtual patients capable of generating human-like voice responses [36,38,44-46]. Notably, 1 study highlighted a more versatile approach, where the virtual patient was designed to provide responses to both text and synthesized speech [42].

Regarding the avatar design of the virtual patient in the included studies, 6 studies used a 3D-embodied virtual patient to enhance realism and immersion for participants [36,38,42,44-46]. For instance, a study [38] integrated a mixed reality tool, allowing participants not only to visually perceive the virtual patient within a digital environment but also to physically interact with a corresponding manikin. This setup enabled the virtual patient to display various injuries, movements, and facial expressions aligned with speech production, respiration patterns, and pain-related vocalizations. Similarly, research by Borg et al [36] implemented a virtual patient embedded within a robotic system. Their robot featured a 3-degree-of-freedom neck and an animated face, facilitating flexible head movements and expressive emotional displays. Mool et al [46] also used a 3D avatar, though it was noted to be largely static within the examination room environment. In contrast, the remaining 9 studies used virtual patients without avatars, relying solely on alternative interaction modalities [33-37,39-41,43,47].

In the 15 included studies, interventions involving participants interacting with GenAI-supported virtual patients lasted less than 10 minutes in 5 cases [33,38-41]. Three studies reported intervention durations exceeding 20 minutes [43-45], while the remaining 5 did not specify the duration [34-37,42]. Moreover, 7 studies featured only a single session, regardless of the intervention length [33,35,38,39,42,44,45].

### Technological Architecture

A total of 13 studies explicitly stated that they used an OpenAI GPT model for generating the virtual patient [33-44,46]. Further, 1 study used a fine-tuned model from a different provider [45]. Yu et al [47] evaluated a broader range of models for their AIPatient system, including Claude models (Haiku, Sonnet variants), GPT-family models (GPT-4 Turbo, GPT-4o, and GPT-3.5 Turbo), and open-source models (DeepSeekV3 671B, Qwen3-32B, and LLaMa-3 70B). All studies specified the foundational GenAI model used. A total of 14 studies provided detailed patient case information as part of the prompt to enhance the virtual patient’s responses [33-35,38,40-47]. For instance, in a study [36], the prompt consisted of a structured patient case description, the previous 10 turns of dialog, and an instruction to generate the next line of conversation. Yu et al [47] took a sophisticated approach, integrating real electronic health record data from the MIMIC-III (Medical Information Mart for Intensive Care III) database and incorporating personality profiles based on the Big Five framework to simulate diverse and realistic patient behaviors. In contrast, 1 study used a simpler prompt, wherein the GenAI was instructed merely to assume the role of a virtual patient with a specified condition (eg, respiratory distress) and engage in dialog with participants acting as nurses, without requiring additional case-specific details [39].

### Educational Strategies

A total of 3 studies explicitly referenced established educational frameworks to inform their design. The study by Kim et al [44] applied an instructional design model for AI education and the technology acceptance model. The study by Luo et al [45] used Kolb’s experiential learning cycle and the Calgary-Cambridge communication framework, while the study by Chinwong et al [43] was grounded in the 5As framework for smoking cessation counseling. The study by Kim et al [44] emphasized the critical role of patient case design, noting that such cases serve as the foundational structure of the curriculum, functioning as learning triggers and providing a platform for students to engage in cognitive processes reflective of physicians’ workplace reasoning. Given the high-fidelity patient simulation and the level of control afforded by GenAI, this innovative approach has the potential to enhance medical education curricula, offering valuable benefits for both students and educators. In contrast, the remaining 12 studies did not specify the application of any educational theory to inform the design of the virtual patient or the overall study methodology [33-42,46,47].

To address the RQ2 concerning the educational effectiveness and learner outcomes of GenAI-supported virtual patients, the following sections analyze the extracted data, which are comprehensively tabulated in Table 3.

**Table 3.** Study design, educational purpose, intervention details, measured outcomes, and primary results of the 15 included studies.

Reference citation	Study design (participants, n)	Educational purpose	Session (duration), n	Outcomes	Validity or reliability test	Results of intervention
[39]	Cross-sectional study (n=12)	Patient communication	1 (10 min)	Ease of use of ChatGPT Learning engagement with ChatGPT Recognition of the usefulness of ChatGPT in clinical education Performance in virtual patient interaction	N/A^a^	Students responded positively to ChatGPT, finding it accessible, engaging, and valuable as a training tool. Those with stronger interaction skills tended to perform better overall. Key communication attributes such as clarity, relevance, and usefulness were linked to stronger outcomes.
[40]	Quasi-experimental study within design (n=6)	Radiographers’ communication skills with patients with claustrophobia	10 (2 min)	Simulation success rate Radiographers’ communication skills	N/A	A total of 60 simulations were conducted, achieving a success rate of 96.7% (58/60). ChatGPT-3.5 exhibited errors in 40% (12/30) of the simulations, while ChatGPT-4 showed no errors. The simulation of clinical scenarios via ChatGPT proves valuable in assessing and testing radiographers’ communication skills, especially in managing patients with claustrophobia during MRI.^b^
[33]	Cross-sectional study (n=106)	Patient history taking	1 (8 minutes)	Quality of OpenAI GPT-4’s role-play capability Completeness of history taking	Interrater reliability, measured by Cohen κ	OpenAI GPT-4 demonstrated highly realistic medical responses, with over 99% deemed plausible. Its evaluations closely matched human ratings overall, though some feedback categories showed weaker agreement where OpenAI GPT-4’s assessments were more detailed or differed from human perspectives.
[34]	Cross-sectional study (n=28)	Patient history taking for medical students	N/A	The performance of OpenAI GPT as a simulated patient Chatbot’s usability	N/A	When questions were explicitly covered by the script (n=502, 60.3%), the GPT-provided answers were mostly based on explicit script information (n=471, 94.4%).
[35]	Quasi-experimental study (intervention group n=35, control group n=110)	Medical students’ interview skills	1 (N/A)	The scores related to medical interviewing in the pre-CC^c^ OSCE^d^ Simulation-based training quality	N/A	Students in the AI^e^-supported group performed better in medical interviews compared to those in the control group. An inverse relationship was noted between their self-reported confidence scores and earlier examination results. Importantly, no safety issues were identified throughout the study.
[41]	Randomized controlled trial (control group n=11, feedback group n=10)	Clinical decision-making in medical students	4 (6 min)	The performance of the participants Clinical reasoning ability	N/A	Medical students showed notable improvement when provided feedback. Initially, both the feedback and control groups performed similarly, confirming balanced assignment. By the end, the feedback group scored significantly higher overall, particularly in creating context and gathering information during clinical decision-making. However, there was no marked progress in their question-focusing skills.
[42]	Cross-sectional study (n=15)	Anesthesia training	1 (N/A)	Students’ perception of the virtual patient: Intuitive User-friendly Accuracy Usability (use the model comfortably) Feasibility	N/A	The survey of 15 anesthetists revealed that the tool was generally well received. It had a median rating of 9 out of 10 for how intuitive and user-friendly it was, and a score of 8 out of 10 for simulating realistic patient responses and behaviors. Furthermore, 87% of the participants reported feeling comfortable using the model, suggesting strong confidence in its design and functionality. It seems the tool succeeded in both usability and clinical accuracy.
[38]	Cross-sectional study (n=24)	Communication training in an emergency (ie, car accident)	1 (6‐10 min)	Perception of voice quality Usability of voice interactions	N/A	The usability assessment of the virtual patient yielded moderately positive feedback, with particularly favorable scores in habitability and likeability. However, the roughly 3-second delay in response time detracted from the fluidity of interactions. MFRs^f^ found it natural to evaluate the virtual patient’s physiological state through verbal questions, but they also noted limitations in the dialog flow, especially the virtual patient’s inability to initiate conversation. A key insight emerged around the potential of using domain-specific prompt engineering to guide responders more effectively during training.
[36]	Quasi-experimental study within design (n=15)	CR^g^ training in rheumatology, comparing a social robotic VP^h^ platform (LLM^i^-enhanced) to a conventional computer-based platform	1 VP case per platform (order counterbalanced). Duration not specified	Virtual patient evaluation Qualitative experiences of clinical reasoning, communication, and emotional skill training	N/A	Quantitative: the social robotic platform was rated significantly higher for authenticity (mean 4.5 vs 3.9, *P*=.04) and overall learning effect (mean 4.4 vs 4.1, *P*=.01). Qualitative: students found the robot superior for training CR, communication, and emotional skills, despite noting technical limitations.
[43]	Single group quasi-experimental, prepost (n=145)	To practice smoking cessation counseling using the 5As^j^ framework with an AI-simulated patient	Practice over 3 weeks (unrestricted frequency or duration), followed by a 2-hour classroom discussion session	Student satisfaction Perceived learning impact Perceived benefits Perceived difficulties	N/A	66% of students were satisfied. Further, 84.4% reported improved understanding. Key benefits included self-assessment and adaptability. Major challenges were technical issues (88.3%) and a lack of AI understanding (58.6%).
[37]	Proof-of-concept observational study (n=28)	To train communication techniques (eg, empathy and motivational interviewing) for mental health encounters using an AI chatbot with real-time feedback	2 chat sessions (20 minutes each) with 2 different AI-simulated patients	Accuracy of AI-generated feedback (expert-evaluated) Participant perception of feedback Change in frequency of communication techniques Perceived benefit for clinical practice	N/A	85.38% of real-time feedback was partially or totally correct. Further, 87.27% of participants found the feedback helpful. A significant increase in the use of targeted techniques was observed from chat 1 to chat 2 (Poisson regression, *P*<.001). Over 80% agreed that the training helped them practice and apply new techniques.
[44]	Single group quasi-experimental, prepost (n=28)	To train health assessment and therapeutic communication skills for patients with acute appendicitis using a GPT-based VP in VR^k^	1 session (1 hour total for interaction and practice)	Quantitative: Usability Perceived virtual learning environment (immersion, usefulness, etc) Self-efficacy in communication Qualitative: Training experiences Accuracy of AI dialogs Safety of AI dialogs Relevance of AI dialogs Readability of AI dialogs	Reliability tested via Cronbach α for all scales: usability (α=.85), perceived virtual learning environment (α=.95), self-efficacy of communication (α=.88).	Self-efficacy in communication increased significantly (pre: 61.57, post: 64.32, *P*=.009). The highest scores were for immersion and function accessibility. Qualitative themes highlighted educational benefits and technical limitations. AI dialog scored highest on readability and lowest on accuracy.
[45]	RCT^l^ (LLMDP^m^ group n=13, control group n=13)	To enhance ophthalmology medical history-taking skills using an LLM-based digital patient system	1 (1 h)	Medical history-taking ability Empathy Student attitudes or satisfaction	N/A	The LLMDP group showed significantly higher MHTA^n^ scores (mean 64.62, SD 9.52) vs control (54.12, SD 8.80), mean difference 10.50 points (95% CI 4.66‐16.33, *P*<.001). The intervention group also demonstrated better empathy. High student satisfaction was reported, highlighting benefits for confidence and cost or time savings.
[46]	Between-groups, mixed methods study (GenAI^o^ groups n=13, ePBLM^p^ group n=13)	To explore what happens when a GenAI-enabled virtual patient is introduced within a PBL^q^ tutorial for history-taking, compared to a legacy multimedia database system (ePBLM)	1 (GenAI groups: 55‐65 min; ePBLM groups: 36‐39 min)	Primary (observational): characterization of student interactions with the patient modality and with each other during history-taking (via descriptive observation of audio-recordings), secondary (survey or quiz) Learner perceptions: 8-item survey (5-point Likert) assessing perceptions of PBL tutorial quality (clinical accuracy, enjoyability, teamwork, etc) Patient history recall: 11-item short-answer quiz assessing recall of patient case information (immediate and 2-week delayed)	Validity: Survey items were judged by a behavioral scientist to be typical of those in other simulated patient studies. Quiz items were verified by 2 faculty members for consistency with the case and readability. Reliability: Quiz grading: first 5 quizzes graded independently by 2 coinvestigators to establish consistency; remaining 52 quizzes graded by 1 investigator. Statistical tests: linear regression (OLS^r^ estimation), 2-tailed with α=.05.	Observational findings: GenAI presented essential case content accurately but occasionally deviated on nonessential content (eg, embellished responses, inconsistent headache history, and unreported marijuana use). GenAI groups took ≈10 minutes longer on history-taking, partly due to collaborative troubleshooting of AI interaction. Students treated the avatar like a sophisticated “question base,” using closed-ended questions and jargon, not realistic patient interviewing. One GenAI group showed more experimental, anthropomorphizing engagement (eg, using the patient’s name and inferring attitude). Survey results: GenAI students rated their experience significantly higher than their prior ePBLM experiences (mean total score 34.38 vs 28.38 pretutorial, *P*=.003). Largest gains were in “simulates clinical experiences accurately” (mean increase of 1.6 points). Quiz results: Immediate recall was near ceiling in both groups (GenAI: 10.10/11; ePBLM: 9.40/11). Delayed recall (2 weeks) decreased significantly in both groups (GenAI: 8.63; ePBLM: 7.94), but the rate of forgetting did not differ by condition (*P*=.052 for condition effect).
[47]	Paired crossover study (n=20 medical students)	To evaluate the fidelity, usability, and educational effectiveness of the AIPatient^s^ system compared to H-SPs^t^ in medical history-taking	4 interactions per student (2 cases×2 modalities: AIPatient and H-SP). Duration not explicitly specified	Primary (system performance): Knowledgebase validity: NER^u^ *F*_1_-score QA^v^ accuracy: percentage correct in EHR^w^-based QA Readability: Flesch Reading Ease, Flesch-Kincaid Grade Level Robustness: accuracy variance with paraphrased questions (ANOVA) Stability: accuracy variance with 32 personality types (ANOVA and data loss percentage) Secondary (user study): Fidelity, usability, and educational effectiveness: measured via 5-point Likert-scale questionnaire Clinical information gathering: OSCE-style checklist Qualitative feedback: semistructured interviews	Intercoder reliability: *F*_1_-score (0.79) for NER gold-standard labels; Cohen κ (0.92) for QA accuracy ratings	AIPatient matched or exceeded H-SPs across most metrics. Significant advantages: emotional realism (4.37 vs 3.74, *P*<.01), technical reliability (4.39 vs 3.79, *P*<.01), improving clinical reasoning skills (4.41 vs 3.97, *P*<.05). OSCE checklist: AIPatient performed comparably or better in supporting clinical reasoning and information elicitation. Qualitative: students found AIPatient emotionally expressive, pedagogically valuable, efficient, consistent, and usable. Identified areas for improvement included verbosity and handling of nonstandard queries.

a N/A: not applicable.

b MRI: magnetic resonance imaging

c Pre-CC: preclinical clerkship.

d OSCE: objective structured clinical examination.

e AI: artificial intelligence.

f MFR: medical first responder.

g CR: clinical reasoning.

h VP: virtual patient.

i LLM: large language model.

j 5A: Ask, Advise, Assess, Assist, Arrange.

k VR: virtual reality.

l RCT: randomized controlled trial.

m LLMDP: large language model-based digital patient.

n MHTA: mental health therapy aide.

o GenAI: generative artificial intelligence.

p ePBLM: electronic problem-based learning.

q PBL: problem-based learning.

r OLS: ordinary least squares.

s AIPatient: artificial intelligence patient.

t H-SP: human-simulated patient.

u NER: named entity recognition.

v QA: question answering.

w EHR: electronic health record.

#### Educational Benefits and Learner Outcomes

Among all 15 included studies, 14 assessed participants’ perceptions of the GenAI-supported virtual patient, examining factors such as usefulness [39], accuracy [42,44], and the authenticity of the patient encounter [36,37,46,47]. Additionally, 11 studies investigated the impact of GenAI-supported virtual patients on participants’ learning outcomes, including performance [33-35,39,41,45,47], communication skills [38,40,44,46], and clinical reasoning ability [36,41,47]. Across multiple studies, GenAI-supported virtual patients demonstrated substantial benefits in health care education. Students rated the tool as accessible, engaging, and pedagogically valuable [39,42,46,47], with advanced models such as ChatGPT-4 achieving high scenario completion rates and error-free performance [33,40]. The simulations yielded highly realistic clinical responses and plausible feedback [33,35], and emotionally expressive interactions were found to be appropriate and contextually accurate [36,47]. Further studies confirmed strong user experiences and authentic communication [38], with improved outcomes in medical interview performance [35,45] and decision-making when feedback mechanisms were integrated [37,41]. The tool also earned high marks for intuitiveness and user comfort [42], enhanced perceptions of authenticity and learning effectiveness over conventional approaches [36,47], and received positive feedback on self-efficacy and the learning environment [44]. Further, 1 study reported that 84.4% of participants perceived an improved understanding of the subject matter [43].

#### Evaluation Designs and Assessment Strategies

A total of 6 studies used an experimental design incorporating a control group to assess the effects of GenAI-supported virtual patients [35,36,41,45-47]. Within these 6 studies, 5 of them compared the GenAI virtual patient with traditional pedagogical methods or a non-AI control [35,36,45-47]. The remaining 1 study used GenAI virtual patients in both the intervention and control groups, but with variations in functionality (conversation-only vs conversation+feedback) [41]. The other 9 studies did not include comparative analyses between GenAI and alternative conditions or conduct prepost intervention comparisons [33,34,37-40,42-44]. None of the studies in this review used a longitudinal design. Regarding data collection approaches, 9 studies relied exclusively on quantitative data [34,35,38-42,45,47], while the remaining 5 studies incorporated both quantitative and qualitative methods [36,37,43,44,46].

#### Design Limitations

Despite their promise, the GenAI-supported virtual patients reviewed exhibit several inherent limitations. Critically, behavioral and emotional complexity remains underdeveloped. While some avatars [42,44,46] incorporated basic movements or facial expressions, these were often simplistic and failed to fully replicate the nuanced nonverbal cues (eg, subtle pain indicators, authentic gaze patterns, and culturally specific gestures) essential for holistic clinical assessment and empathy training. For instance, Mool et al [46] noted that their 3D avatar was largely static within the examination room environment, with no description of physical movement. Emotional responsiveness was largely superficial, relying on prescripted ranges or simplistic vocalizations [38,42], rather than dynamically adapting emotional states based on learner interaction or physiological parameters. Furthermore, adaptability beyond dialog was constrained; virtual patients struggled to simulate evolving physical symptoms (eg, changing breath sounds and deteriorating vital signs) or accurately respond to physical examination maneuvers performed by learners within simulations. Memory and longitudinal consistency across interactions were absent, preventing virtual patients from recalling prior conversations or learner actions to build continuity. Finally, the underlying GenAI models (predominantly ChatGPT variants) introduced inherent biases and factual inaccuracies in medical content, alongside potential cultural insensitivities, raising concerns about the reliability and safety of the clinical scenarios portrayed. These limitations collectively restrict the virtual patients’ ability to fully mirror the dynamism and unpredictability of real patient encounters.

In terms of limitations in research methodology, 13 of the 15 studies did not report the evaluation of the reliability or validity of their assessment instruments [33-43,45,46]. Further, 2 studies explicitly tested reliability [44,47]. Furthermore, 9 studies used designs without a control group and did not measure the same variables before and after the intervention [33,34,36-40,42,45,47], making it unclear whether the reported outcomes resulted from the intervention itself or differed across other conditions.

## Discussion

### Principal Findings

The current systematic review provides a comprehensive synthesis of the emerging evidence on GenAI-supported virtual patients in health care education, focusing on implementation characteristics, educational impact, and methodological considerations. Building on the descriptive findings presented in the Results section, the following sections offer analytical comparisons across study designs, AI modalities, and educational purposes, and map identified limitations to established learning theories to guide future development.

### Synthesis of Findings by Study Design, AI Modality, and Educational Purpose

When examining the evidence base by study design, a clear pattern emerges regarding the strength of causal inferences that can be drawn. The 3 RCTs provide the most robust evidence, demonstrating significant improvements in clinical decision-making [41], ophthalmology history-taking skills [45], and medical history-taking performance [47] attributable to GenAI-supported virtual patient interventions. In contrast, the cross-sectional and quasi-experimental studies, while consistently reporting positive user perceptions and self-reported skill gains [33,34,38,39,42,46], are more susceptible to enthusiasm bias and cannot establish definitive causal links between the intervention and learning outcomes. This methodological gradient underscores the need for more rigorous, controlled trials to move the field beyond proof-of-concept toward evidence-based practice.

Critically, the overall evidence base is characterized by small sample sizes, brief intervention durations (often single sessions under 10 minutes), and a near-complete absence of longitudinal follow-up. Consequently, while the studies demonstrate that GenAI-supported virtual patients are feasible and acceptable to learners, claims regarding their educational effectiveness must remain preliminary. The current findings support feasibility and acceptability more strongly than demonstrable learning gains or transfer to clinical practice.

Analysis by AI modality reveals differential alignment with educational objectives. Studies using embodied, voice-based virtual patients [36,38,44-46] predominantly targeted communication skills and emotional realism as primary outcomes. For example, Borg et al [36] found that a social robotic platform with 3D embodiment was rated significantly higher for authenticity and emotional skill training compared to a conventional computer-based platform, suggesting that physical presence and nonverbal cues may be particularly valuable for interpersonal skill development. In contrast, text-based virtual patient studies [33-35,37,39-41,43,47] more frequently targeted clinical reasoning, diagnostic accuracy, and history-taking performance. Yu et al [47] directly compared their text-based AI patient to human-simulated patients and found comparable or superior support for clinical reasoning, indicating that for cognitive skills such as information gathering and diagnostic thinking, sophisticated text-based interactions may be as effective as, or more effective than, human simulations. This modality-outcome alignment has practical implications for instructional design: educators should select or design GenAI virtual patient platforms based on the specific competencies they aim to develop.

When considering educational purpose, studies targeting communication skills [38,40,44,46] consistently reported improvements in learner empathy, interviewing technique, and patient interaction quality. For instance, Maquilón et al [38] found that medical first responders rated the virtual patient’s voice interactions as natural for assessing physiological state, while Mool et al [46] observed that some students anthropomorphized the avatar, using the patient’s name and inferring attitudes—behaviors indicative of authentic communication practice. Studies focused on clinical reasoning [36,41,47] demonstrated gains in diagnostic accuracy, information gathering, and decision-making, with Brügge et al [41] showing that feedback-enhanced interactions led to significantly higher clinical reasoning scores. This pattern suggests that GenAI-supported virtual patients can be effectively tailored to specific educational objectives, with modality and design features aligning with targeted learning outcomes.

### Theoretical Implications of Design Limitations

From the perspective of experiential learning theory [48], the lack of longitudinal consistency and evolving patient states prevents learners from engaging in the full cycle of concrete experience, reflective observation, abstract conceptualization, and active experimentation across repeated encounters. Without memory across sessions, students cannot build upon prior interactions or observe the consequences of their clinical decisions over time—a core component of developing clinical expertise. This limitation suggests that future GenAI virtual patient designs should incorporate persistent patient states and longitudinal case progression to support complete experiential learning cycles.

Cognitive load theory [49,50] provides another lens for interpreting current limitations. The technical glitches, inconsistent responses, and need to troubleshoot AI interactions observed in several studies [38,43,46] impose extraneous cognitive load, diverting mental resources away from the germane load essential for schema construction and clinical reasoning development. Reducing technical unpredictability through more robust prompt engineering and model selection, as demonstrated by Yu et al [47], could minimize extraneous load and optimize cognitive resources for learning.

Furthermore, the absence of adaptive emotional and behavioral complexity limits opportunities for the sustained, feedback-driven practice necessary to refine complex interpersonal skills. Well-established principles of skill acquisition emphasize the importance of repeated engagement with authentic tasks, immediate feedback, and progressive challenge—elements that current GenAI virtual patients only partially provide. Incorporating dynamically adjusting emotional states based on learner interactions, informed by frameworks such as the Big Five personality model used by Yu et al [47], could better support the deliberate practice of communication and empathy.

### Implications in Practice and Research

The implementation of GenAI-supported virtual patients in health care education carries significant practical implications. Educators and curriculum designers must carefully consider the technological infrastructure required to support diverse interaction modalities—text, voice, and hybrid formats—to cater to varied learning preferences and replicate realistic clinical scenarios. The integration of dynamic, AI-generated responses demands ongoing technical oversight and periodic updates to ensure the content remains accurate and relevant. Furthermore, the relatively brief interaction sessions observed in this study suggest that more extended, immersive simulations are needed to mirror the complexity of real-world clinical practice. This calls for interdisciplinary collaborations between educators, clinicians, and technology developers to ensure that practical implementations not only leverage the technical advantages of GenAI but also align with educational objectives and clinical standards.

The finding that only 3 of the 15 included studies were explicitly grounded in established educational frameworks highlights an important area for future research. The limited adoption of frameworks indicates that many current approaches are primarily driven by technological innovation rather than pedagogical insight. By integrating robust theoretical perspectives, the design of GenAI-supported virtual patients can be enhanced to facilitate richer, more targeted learning experiences that promote the development of clinical reasoning and decision-making skills. Furthermore, theoretical models can help in formulating clear hypotheses about how these advanced simulations influence learner outcomes, guiding both the design of interventions and the evaluation of their effectiveness [51]. Encouraging further interdisciplinary studies that explicitly link technology-driven interventions with established learning theories will be essential in building a more comprehensive understanding of how these innovations can transform health care education.

For research design, only 5 of the 15 studies incorporated experimental designs with control groups, allowing for direct comparisons between traditional simulation methods and novel AI-driven interventions [35,36,41,45,47]. This experimental rigor has been crucial in establishing a baseline understanding of the potential advantages of GenAI-enhanced simulations, especially in relation to improving communication skills and clinical reasoning. The remaining 10 studies typically used within-group designs with mixed methods or solely quantitative approaches, providing insights into user perceptions and immediate learning outcomes [33,34,37-40,42-44,46]. As highlighted in the synthesis above, the current evidence base supports feasibility and acceptability more strongly than conclusive educational effectiveness. Future research must therefore prioritize larger, rigorous trials with long-term follow-up and objective measures of skill transfer to clinical practice.

To provide a more balanced perspective, the potential benefits of GenAI-supported virtual patients must be considered alongside their well-documented risks and ethical challenges. Our findings regarding positive learner perceptions and skill development exist within a context of significant technological limitations. Key concerns include algorithmic bias inherent in the training data of LLMs, which could reinforce stereotypes or lead to culturally insensitive patient portrayals, thereby misinforming clinical empathy and communication [52,53] Data privacy and security present another critical challenge, as sensitive health information used in prompt engineering or generated during simulated dialogs requires robust safeguards to comply with regulations such as HIPAA (Health Insurance Portability and Accountability Act) or GDPR (General Data Protection Regulation) [54,55]. Most critically, the tendency of GenAI models to generate plausible but incorrect or fabricated information—known as “hallucinations”—poses a direct threat to clinical accuracy [56]. In a health care education setting, an AI virtual patient providing factually wrong symptoms, pathophysiology, or treatment responses could seriously compromise foundational medical knowledge and patient safety. Therefore, the implementation of these tools necessitates rigorous validation frameworks, ongoing human oversight, and clear institutional guidelines to mitigate these risks, ensuring that innovation in simulation does not come at the cost of pedagogical integrity or ethical responsibility.

### Integration of Methodological Quality

The risk of bias assessment conducted for this review underscores a critical consideration when interpreting its findings. While 3 RCTs demonstrated relatively strong methodological rigor (low to low-moderate risk) [57], most of the evidence derives from quasi-experimental and cross-sectional studies with moderate to high risk of bias [58]. This methodological landscape indicates that the current evidence base is still in a formative, proof-of-concept stage [59]. The prevalent limitations—such as small sample sizes, absence of control groups, lack of blinding, and reliance on unvalidated or self-reported outcomes—suggest that the positive results regarding user acceptance, perceived learning, and skill improvement should be viewed as promising preliminary signals rather than conclusive evidence of efficacy [60-63]. These design weaknesses increase the risk of overestimating positive effects due to confounding, measurement bias, or participant enthusiasm for novel technology [64,65]. Therefore, the synthesized findings, particularly those related to educational impact, must be interpreted with appropriate caution. This appraisal directly informs the primary recommendation of this review: future research must prioritize methodological robustness, including larger-scale randomized designs with active control groups [66], longitudinal follow-up [67], and the use of validated, objective outcome measures [65] to establish a more definitive evidence base for the educational effectiveness of GenAI-supported virtual patients.

Furthermore, our analysis identified variable reporting of ethical considerations—such as institutional review board approval and data security measures—across the included studies. While systematic reviews themselves do not require ethical approval, transparency in primary research is a cornerstone of methodological rigor and trustworthiness [68]. Moving forward, consistent and explicit ethical reporting should be considered a standard in this domain, especially when research involves simulated patient interactions and learner data, to ensure the credibility and safe translation of findings into educational practice [53,54].

### Limitations

This review has several limitations that should be addressed when interpreting its findings. First, while our systematic search was comprehensive across several databases, the inclusion criteria limited the review to English-language studies, potentially excluding relevant research published in other languages or in alternative repositories. Second, many of the primary studies included in this review are constrained by methodological limitations, such as small sample sizes, short simulation durations, and an overreliance on self-reported outcomes, which restrict the generalizability of the findings. Third, the heterogeneity in study designs and assessment tools across the interventions complicates direct comparisons and synthesis of outcomes. Furthermore, our search was limited to published, peer-reviewed literature and did not include gray literature sources such as clinical trial registries or preprint servers. While this decision aligns with standard systematic review practices, it is particularly consequential in a rapidly evolving field where early innovations often first appear as preprints or technical reports. Consequently, the review may not capture the most recent developments or emerging design approaches that have not yet undergone formal peer review. Future updates to this review should consider expanding the search to include gray literature as the evidence base matures. Lastly, the current review only included 15 papers; this limited number of studies may not capture the full spectrum of innovative practices and outcomes in this rapidly evolving field, thereby constraining the robustness of the conclusions drawn.

### Conclusions

In summary, this review confirms that GenAI-supported virtual patients offer notable advances in adaptability and interactivity. This study is innovative as it constitutes the first dedicated synthesis of this specific technological application in health care education. It differs from prior reviews of virtual patients or GenAI by focusing exclusively on their intersection, thereby isolating the unique capabilities and challenges introduced by GenAI. The review brings to the field a foundational framework that classifies key design dimensions, evaluates educational impact, and identifies critical gaps, setting a clear agenda for subsequent research. The real-world implications are significant: for educators and technologists, it provides an evidence-based roadmap for developing more effective, theory-informed simulations; for institutions, it highlights the practical considerations and potential transformative value of integrating these tools to modernize clinical skills training and address scalability in health care education.

## Supplementary material

10.2196/82756 Multimedia Appendix 1 Synthesis Without Meta-analysis (SWiM) reporting items.

10.2196/82756 Multimedia Appendix 2 Search strategy.

10.2196/82756 Checklist 1 PRISMA 2020 expanded checklist.

## Abbreviations

AI: artificial intelligence
GDPR: General Data Protection Regulation
GenAI: generative artificial intelligence
GRADE: Grading of Recommendations, Assessment, Development, and Evaluation
HIPAA: Health Insurance Portability and Accountability Act
JBI: Joanna Briggs Institute
LLM: large language model
MeSH: Medical Subject Headings
MIMIC-III: Medical Information Mart for Intensive Care III
PRISMA: Preferred Reporting Items for Systematic Reviews and Meta-Analyses
PRISMA-S: Preferred Reporting Items for Systematic Reviews and Meta-Analyses Literature Search Extension
RCT: randomized controlled trial
RQ: research question
