# Supplementary material for: GenAI-Supported Virtual Patients in Health Care Education: Systematic Review
Source: J Med Internet Res. 2026 May 7;28:e82756. doi: 10.2196/82756 (PMC13152703; doi:10.2196/82756)
Supplement: Multimedia Appendix 1 [file jmir-v28-e82756-s001.docx]

| **Synthesis Without Meta-analysis (SWiM) reporting items** | | | |
| --- | --- | --- | --- |
| **SWiM reporting item** | **Item description** | **Page in manuscript where item is reported** | **Other*** |
| **Methods** | | | |
| 1 Grouping studies for synthesis | 1a) Provide a description of, and rationale for, the groups used in the synthesis (e.g., groupings of populations, interventions, outcomes, study design) | Methods – Synthesis Methods (p. 8): “The extracted data were summarized and organized to address the review’s research questions. Findings were structured thematically to describe: (1) the implementation characteristics (design, technology, educational strategies) of GenAI‑supported virtual patients, and (2) their evaluation and educational impact (benefits, outcomes, methodological approaches, and limitations).” |  |
|  | 1b) Detail and provide rationale for any changes made subsequent to the protocol in the groups used in the synthesis | No changes; protocol registered at OSF (link in Methods, p. 5) |  |
| 2 Describe the standardised metric and transformation methods used | Describe the standardised metric for each outcome. Explain why the metric(s) was chosen, and describe any methods used to transform the intervention effects, as reported in the study, to the standardised metric, citing any methodological guidance consulted | Not applicable. Methods – Effect Measures (p. 8): “Current review synthesized findings narratively, thus no common effect measures were pooled across studies due to significant heterogeneity in study designs, interventions, and outcome measures.” |  |
| 3 Describe the synthesis methods | Describe and justify the methods used to synthesise the effects for each outcome when it was not possible to undertake a meta-analysis of effect estimates | Methods – Synthesis Methods (p. 8): “A meta‑analysis was not feasible due to the limited number of studies and substantial heterogeneity in interventions, populations, and outcome measures. Therefore, a narrative synthesis was conducted. The extracted data were summarized and organized to address the review’s research questions. Findings were structured thematically … The synthesis also integrates a discussion of the methodological quality and risk of bias of the included studies.” |  |
| 4 Criteria used to prioritise results for summary and synthesis | Where applicable, provide the criteria used, with supporting justification, to select the particular studies, or a particular study, for the main synthesis or to draw conclusions from the synthesis (e.g., based on study design, risk of bias assessments, directness in relation to the review question) | Methods – Study Risk of Bias Assessment (p. 8): formal JBI assessment performed; results considered when interpreting overall strength and validity of evidence. Methods – Synthesis Methods (p. 8): “The synthesis also integrates a discussion of the methodological quality and risk of bias of the included studies.” All eligible studies were included in the synthesis, but risk of bias and study design were used to weight conclusions in the Discussion. |  |
| 5 Investigation of heterogeneity in reported effects | State the method(s) used to examine heterogeneity in reported effects when it was not possible to undertake a meta-analysis of effect estimates and its extensions to investigate heterogeneity | Methods – Synthesis Methods (p. 8): heterogeneity was assessed descriptively (no statistical methods). The synthesis was narrative, and heterogeneity is noted in the text (e.g., “due to the limited number of studies and substantial heterogeneity in designs, interventions, and outcome measures”). |  |
| 6 Certainty of evidence | Describe the methods used to assess certainty of the synthesis findings | Methods – Certainty Assessment (p. 8): “A formal assessment of the certainty of the body of evidence (e.g., GRADE) was not conducted for this narrative review, as its primary aim was to map and characterize an emerging field rather than to estimate a pooled treatment effect.” Methods – Study Risk of Bias Assessment (p. 8): JBI checklists were used to assess methodological quality; results were considered when interpreting the overall strength and validity of the evidence. |  |
| 7 Data presentation methods | Describe the graphical and tabular methods used to present the effects (e.g., tables, forest plots, harvest plots). Specify key study characteristics (e.g., study design, risk of bias) used to order the studies, in the text and any tables or graphs, clearly referencing the studies included | Methods – Synthesis Methods (p. 8): “Findings were structured thematically …” Results (pp. 9–12): PRISMA flow diagram (Figure 1); Table 2 (Risk of Bias) orders studies by design and risk; Multimedia Appendices 2 and 3 present detailed study characteristics and outcomes. |  |
| **Results** | | | |
| 8 Reporting results | For each comparison and outcome, provide a description of the synthesised findings, and the certainty of the findings. Describe the result in language that is consistent with the question the synthesis addresses, and indicate which studies contribute to the synthesis | Results – Results of syntheses (pp. 10–12): RQ1 and RQ2 answered with narrative descriptions, counts, and citations to individual studies. |  |
| **Discussion** | | | |
| 9 Limitations of the synthesis | Report the limitations of the synthesis methods used and/or the groupings used in the synthesis, and how these affect the conclusions that can be drawn in relation to the original review question | Discussion – Limitations (p. 17): addresses restrictions to English‑language and peer‑reviewed literature, exclusion of grey literature, small number of studies, heterogeneity, and preliminary nature of the evidence; explains how these limit conclusions about educational effectiveness. |  |
