# Supplementary material for: GenAI-Supported Virtual Patients in Health Care Education: Systematic Review
Source: J Med Internet Res. 2026 May 7;28:e82756. doi: 10.2196/82756 (PMC13152703; doi:10.2196/82756)
Supplement: Multimedia Appendix 2 [file jmir-v28-e82756-s002.docx]

| **Databases & Platforms** | • CINAHL (via EBSCOhost) |
| --- | --- |
|  | • MEDLINE (via EBSCOhost) |
|  | • Embase (via Elsevier) |
|  | • Scopus (via Elsevier) |
|  | • Web of Science Core Collection (via Clarivate) |
| **Multi-database Searching** | Not applicable. Each database was searched individually via its native platform. |
| **Date of Search & Coverage** | Final search executed on 19th March 2026. The search covered records from 1966 to 19th March 2026. |
| **Limits / Filters** | The initial search was limited for articles that published in English. Only peer-reviewed articles were considered during screening. |
| **Other Sources** | Citation Searching: Reference lists of included full-text articles were manually reviewed. |
|  | Grey Literature: Not searched. |

| **Search syntax for CINAHL via MyEBSCO** | | | |
| --- | --- | --- | --- |
| Line | Boolean | Query | Field |
| S1 | N/A | MH "Patient Simulation+" | All fields |
| S2 | OR | XB ("virtual patient" OR "simulated patient" OR "standardized patient" OR "virtual standardized patient" OR "digital patient*") | Title and Abstract |
| S3 | OR | XB ("patient simulator" OR "computer-based patient simulation" OR "computer-based simulation" OR "clinical simulation" OR "high-fidelity simulation" OR "patient simulation") | Title and Abstract |
| S4 | OR | XB ("virtual case" OR "simulated case") | Title and Abstract |
| S5 | OR | XB ("avatar patient" OR "interactive patient") | Title and Abstract |
| S6 | OR | XB ("computerized patient" OR "digital clinical scenario") | Title and Abstract |
| S7 | OR | XB ("virtual clinical encounter" OR "simulated clinical encounter") | Title and Abstract |
| S8 | OR | XB ("digital twin" OR "virtual consultation" OR "simulated consultation*") | Title and Abstract |
| S9 | AND | MH "Generative Artificial Intelligence+" | All fields |
| S10 | OR | XB ("large language model*" OR LLM OR LLMs) | Title and Abstract |
| S11 | OR | XB ("generative artificial intelligence" OR "generative AI" OR GenAI) | Title and Abstract |
| S12 | OR | XB (ChatGPT OR GPT OR "GPT-3" OR "GPT-4" OR "GPT-3.5" OR "generative pre-trained transformer*") | Title and Abstract |
| S13 | OR | XB ("conversational AI" OR "chatbot" OR "AI chatbot" OR "intelligent virtual agent" OR "conversational agent" OR "dialogue system*") | Title and Abstract |
| S14 |  | (S1 OR S2 OR S3 OR S4 OR S5 OR S6 OR S7 OR S8) AND (S9 OR S10 OR S11 OR S12 OR S13) | Combine sets |
| S15 |  | S14 | Limit to English language (LA English) |
| S16 |  | S15 | Limit to peer‑reviewed articles (PEER(yes)) |

| **Search syntax for MEDLINE via MyEBSCO** | | | |
| --- | --- | --- | --- |
| Line | Boolean | Query | Field |
| S1 | N/A | MH "Patient Simulation+" | All fields |
| S2 | OR | XB ("virtual patient" OR "simulated patient" OR "standardized patient" OR "virtual standardized patient" OR "digital patient*") | Title and Abstract |
| S3 | OR | XB ("patient simulator" OR "computer-based patient simulation" OR "computer-based simulation" OR "clinical simulation" OR "high-fidelity simulation" OR "patient simulation") | Title and Abstract |
| S4 | OR | XB ("virtual case" OR "simulated case") | Title and Abstract |
| S5 | OR | XB ("avatar patient" OR "interactive patient") | Title and Abstract |
| S6 | OR | XB ("computerized patient" OR "digital clinical scenario") | Title and Abstract |
| S7 | OR | XB ("virtual clinical encounter" OR "simulated clinical encounter") | Title and Abstract |
| S8 | OR | XB ("digital twin" OR "virtual consultation" OR "simulated consultation*") | Title and Abstract |
| S9 | AND | MH "Generative Artificial Intelligence+" | All fields |
| S10 | OR | XB ("large language model*" OR LLM OR LLMs) | Title and Abstract |
| S11 | OR | XB ("generative artificial intelligence" OR "generative AI" OR GenAI) | Title and Abstract |
| S12 | OR | XB (ChatGPT OR GPT OR "GPT-3" OR "GPT-4" OR "GPT-3.5" OR "generative pre-trained transformer*") | Title and Abstract |
| S13 | OR | XB ("conversational AI" OR "chatbot" OR "AI chatbot" OR "intelligent virtual agent" OR "conversational agent" OR "dialogue system*") | Title and Abstract |
| S14 |  | (S1 OR S2 OR S3 OR S4 OR S5 OR S6 OR S7 OR S8) AND (S9 OR S10 OR S11 OR S12 OR S13) | Combine sets |
| S15 |  | S14 | Limit to English language (LA English) |
| S16 |  | S15 | Limit to peer‑reviewed articles (PEER(yes)) |

| **Search syntax for Embase via Elsevier** | | | |
| --- | --- | --- | --- |
| Line | Boolean | Query | Field |
| S1 | OR | ("virtual patient" OR "simulated patient" OR "standardized patient" OR "virtual standardized patient" OR "digital patient*"):ti,ab | Title and Abstract |
| S2 | OR | ("patient simulator" OR "computer-based patient simulation" OR "computer-based simulation" OR "clinical simulation" OR "high-fidelity simulation" OR "patient simulation"):ti,ab | Title and Abstract |
| S3 | OR | ("virtual case" OR "simulated case"):ti,ab | Title and Abstract |
| S4 | OR | ("avatar patient" OR "interactive patient"):ti,ab | Title and Abstract |
| S5 | OR | ("computerized patient" OR "digital clinical scenario"):ti,ab | Title and Abstract |
| S6 | OR | ("virtual clinical encounter" OR "simulated clinical encounter"):ti,ab | Title and Abstract |
| S7 | OR | ("digital twin" OR "virtual consultation" OR "simulated consultation*"):ti,ab | Title and Abstract |
| S8 | OR | ("large language model*" OR LLM OR LLMs):ti,ab | Title and Abstract |
| S9 | OR | ("generative artificial intelligence" OR "generative AI" OR GenAI):ti,ab | Title and Abstract |
| S10 | OR | (ChatGPT OR GPT OR "GPT-3" OR "GPT-4" OR "GPT-3.5" OR "generative pre-trained transformer*"):ti,ab | Title and Abstract |
| S11 | OR | ("conversational AI" OR "chatbot" OR "AI chatbot" OR "intelligent virtual agent" OR "conversational agent" OR "dialogue system*"):ti,ab | Title and Abstract |
| S12 |  | (S1 OR S2 OR S3 OR S4 OR S5 OR S6 OR S7) AND (S8 OR S9 OR S10 OR S11) | Combine sets |
| S13 |  | S12 | Limit to English language (English only) |
| S14 |  | S13 | Limit to peer‑reviewed articles (peer‑reviewed) |

| **Search syntax for Scopus via Elsevier** | | | |
| --- | --- | --- | --- |
| Line | Boolean | Query | Field |
| S1 | OR | TITLE-ABS-KEY("virtual patient" OR "simulated patient" OR "standardized patient" OR "virtual standardized patient" OR "digital patient*") | Title, Abstract, Keywords |
| S2 | OR | TITLE-ABS-KEY("patient simulator" OR "computer-based patient simulation" OR "computer-based simulation" OR "clinical simulation" OR "high-fidelity simulation" OR "patient simulation") | Title, Abstract, Keywords |
| S3 | OR | TITLE-ABS-KEY("virtual case" OR "simulated case") | Title, Abstract, Keywords |
| S4 | OR | TITLE-ABS-KEY("avatar patient" OR "interactive patient") | Title, Abstract, Keywords |
| S5 | OR | TITLE-ABS-KEY("computerized patient" OR "digital clinical scenario") | Title, Abstract, Keywords |
| S6 | OR | TITLE-ABS-KEY("virtual clinical encounter" OR "simulated clinical encounter") | Title, Abstract, Keywords |
| S7 | OR | TITLE-ABS-KEY("digital twin" OR "virtual consultation" OR "simulated consultation*") | Title, Abstract, Keywords |
| S8 | OR | TITLE-ABS-KEY("large language model*" OR LLM OR LLMs) | Title, Abstract, Keywords |
| S9 | OR | TITLE-ABS-KEY("generative artificial intelligence" OR "generative AI" OR GenAI) | Title, Abstract, Keywords |
| S10 | OR | TITLE-ABS-KEY(ChatGPT OR GPT OR "GPT-3" OR "GPT-4" OR "GPT-3.5" OR "generative pre-trained transformer*") | Title, Abstract, Keywords |
| S11 | OR | TITLE-ABS-KEY("conversational AI" OR "chatbot" OR "AI chatbot" OR "intelligent virtual agent" OR "conversational agent" OR "dialogue system*") | Title, Abstract, Keywords |
| S12 |  | (S1 OR S2 OR S3 OR S4 OR S5 OR S6 OR S7) AND (S8 OR S9 OR S10 OR S11) | Combine sets |
| S13 |  | S12 | Limit to English language (after search) |
| S14 |  | S13 | Limit to peer‑reviewed articles (after search) |

| **Search syntax for Web of Science via Clarivate** | | | |
| --- | --- | --- | --- |
| Line | Boolean | Query | Field |
| S1 | OR | TS=("virtual patient" OR "simulated patient" OR "standardized patient" OR "virtual standardized patient" OR "digital patient*") | Topic (Title, Abstract, Keywords) |
| S2 | OR | TS=("patient simulator" OR "computer-based patient simulation" OR "computer-based simulation" OR "clinical simulation" OR "high-fidelity simulation" OR "patient simulation") | Topic |
| S3 | OR | TS=("virtual case" OR "simulated case") | Topic |
| S4 | OR | TS=("avatar patient" OR "interactive patient") | Topic |
| S5 | OR | TS=("computerized patient" OR "digital clinical scenario") | Topic |
| S6 | OR | TS=("virtual clinical encounter" OR "simulated clinical encounter") | Topic |
| S7 | OR | TS=("digital twin" OR "virtual consultation" OR "simulated consultation*") | Topic |
| S8 | OR | TS=("large language model*" OR LLM OR LLMs) | Topic |
| S9 | OR | TS=("generative artificial intelligence" OR "generative AI" OR GenAI) | Topic |
| S10 | OR | TS=(ChatGPT OR GPT OR "GPT-3" OR "GPT-4" OR "GPT-3.5" OR "generative pre-trained transformer*") | Topic |
| S11 | OR | TS=("conversational AI" OR "chatbot" OR "AI chatbot" OR "intelligent virtual agent" OR "conversational agent" OR "dialogue system*") | Topic |
| S12 |  | (S1 OR S2 OR S3 OR S4 OR S5 OR S6 OR S7) AND (S8 OR S9 OR S10 OR S11) | Combine sets |
| S13 |  | S12 | Limit to English language (after search) |
| S14 |  | S13 | Limit to peer‑reviewed articles (after search) |
